# The first finding of *Hyalomma rufipes* in Poland in 2024: the promising start of a citizen science project

**DOI:** 10.1186/s13071-025-07022-4

**Published:** 2025-09-24

**Authors:** Wiktoria Romanek, Dorota Dwużnik-Szarek, Dagmara Wężyk, Wiktoria Małaszewicz, Mohammed Alsarraf, Anna W. Myczka, Anna Bajer

**Affiliations:** Department of Eco-Epidemiology of Parasitic Diseases, Institute of Developmental Biology and Biomedical Sciences, Faculty of Biology, University of Warsaw, Miecznikowa 1, 02-096 Warsaw, Poland

**Keywords:** *Hyalomma rufipes*, *Hyalomma marginatum*, Poland, Citizen science

## Abstract

**Background:**

*Hyalomma* spp. ticks play a crucial role as vectors for the Crimean–Congo hemorrhagic fever virus. *Hyalomma* spp. larvae and nymphs are transported via migratory birds to temperate regions of Europe from Africa, the Middle East, and Mediterranean areas. Recently, the emergence of adult ticks has been documented in numerous countries where they were previously not reported. This study aimed to monitor the potential occurrence of *Hyalomma* spp. ticks in Poland using a citizen science project.

**Methods:**

A dedicated website was created through which volunteers could submit photos of unusual ticks. Between April and November 2024, more than 500 online submissions containing tick photos were received, 11 of which were identified as *Hyalomma* spp. ticks.

In addition, we received 65 parcels containing ticks, including four *Hyalomma* spp. ticks (of 11 online submissions). Amplification and sequencing of the partial mitochondrial cytochrome c oxidase subunit I gene (*cox 1*) was successfully performed for all received specimens.

**Results:**

*Hyalomma* spp. ticks were recorded in different regions of Poland, and most records were from the Greater Poland and Silesia regions, in Western and Southwestern Poland, respectively. Two of the identified specimens were morphologically and molecularly characterized as *Hyalomma rufipes*, while another two were identified as *Hyalomma marginatum*.

**Conclusions:**

The citizen science project enabled the confirmation of occurrence of adult *Hyalomma* spp. ticks in Poland, identifying a new hazard for human and animal health.

**Graphical Abstract:**

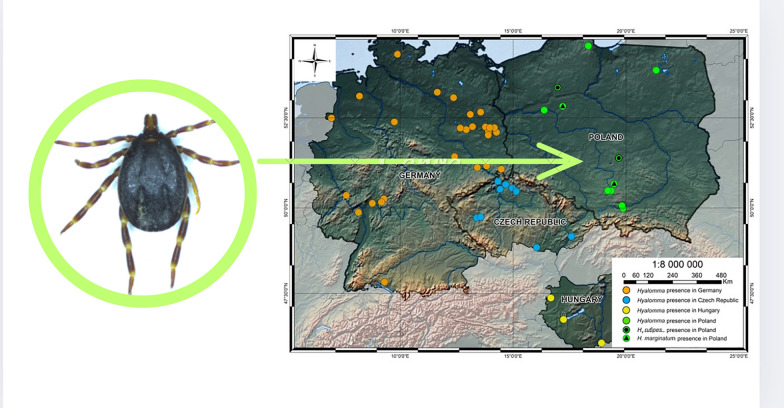

**Supplementary information:**

The online version contains supplementary material available at 10.1186/s13071-025-07022-4.

## Background

In recent years, there has been growing interest in the expansion of tick species into new geographical areas, including ticks of genus *Hyalomma* [1–3]. The observed spread of thermophilic tick species is likely driven primarily by climate change [4, 5]. *Hyalomma* spp. (Acari: *Ixodidae*) are a good example of thermophilic ticks that thrive in warm, dry environments such as deserts, semi-deserts, and pastures across southern Europe, the Middle East, Africa, and Asia [6, 7]. The early developmental stages of *Hyalomma* spp. ticks (larvae and nymphs) typically feed on small mammals and birds, while adult ticks are more commonly found on larger hosts such as livestock, wild ungulates, and horses [8].

Historically, the spread of *Hyalomma* spp. ticks to temperate regions has been limited by unfavorable climatic conditions, including low temperatures and high humidity in spring, which prevented the development of adult ticks from imported larvae and nymphs [9]. However, in recent years new reports on adult questing or feeding *Hyalomma* spp. ticks appeared in regions previously considered outside their natural range—in central and northern Europe [10–13].

Migratory birds play a crucial role in the long-distance dispersal of juvenile *Hyalomma* spp. ticks into new regions [14, 15]. Extensive studies conducted in southern and eastern Europe, involving thousands of migratory birds from Africa, have identified *Hyalomma* larvae and nymphs as ticks most commonly infesting birds [14, 16–19]. Similarly, juvenile *Hyalomma* spp. have been observed on migratory birds, albeit at a significantly lower prevalence in western and northern Europe, including the UK [20], Sweden [21], Norway [19], and the Netherlands [22].

Ticks of genus *Hyalomma* are responsible for the transmission of various pathogens, including viruses, bacteria, and protozoa [23, 24]. One of the most important pathogens transmitted by these ticks is Crimean–Congo hemorrhagic fever virus (CCHFV), causing Crimean–Congo hemorrhagic fever (CCHF), a disease of high fatality in humans [25]. The main threat of the spread of *Hyalomma* spp. ticks in Europe is strictly associated with its vector role for CCHFV. In recent years, there has been an increase in CCHF cases in Spain, raising concerns about the potential spread of the disease in Europe [26]. The CCHFV has been detected in *Hyalomma* spp. ticks from regions such as Kosovo [27] or Spain [28], linking the rise in cases to the presence of these ticks [29]. In addition, *Hyalomma* spp*.* ticks are also implicated in the transmission of several bacterial agents such as *Anaplasma* or *Rickettsia* spp. [30, 31].

Poland is a large country in the central and Northeastern region of Europe and to date, only few reports on *Hyalomma marginatum* occurrence in Poland are available. Four adult *H. marginatum* ticks (four ticks in total) were collected in Bytom (Silesia, Southern Poland) over an 8-year interval (1935 and 1943) (Fig. ?). After World War II, adult *Hyalomma* spp. ticks were not recorded in Poland. However, Siuda and Dutkiewicz (1979), and Nowak and Solarz (2013) reported juvenile *H. marginatum* collected from migrating birds, namely the Yellow Wagtail** (Motacilla flava)** and Sedge Warbler (*Acrocephalus schoenobaenus)*, respectively [32, 33]. Despite intensive field studies on *Ixodes ricinus* and *Dermacentor reticulatus* ticks in numerous regions of the country [34–37], no questing adult *Hyalomma* spp. were recorded to date. However, as these studies primarily relied on flagging and dragging methods, which are generally less effective in detecting adult *Hyalomma* ticks due to their active, hunter-like host-seeking behavior, the absence of findings may have reflected methodological limitations rather than true absence.

In recent years, a number of citizen science projects were set up to monitor tick occurrence and expansion in different regions of the world [13, 22, 38]. Citizen science is a scientific approach that engages the public in research activities, where volunteers contribute to data collection in collaboration with professional researchers. In Europe, citizen science has played a crucial role in monitoring tick populations, particularly in tracking the distribution of species such as *D. reticulatus* [38, 39] or *Hyalomma* spp. [5, 13]. Through online platforms and mobile applications, members of the public can submit detailed reports of tick encounters, which enable genus/species identification, developmental stage determination, and geographical distribution mapping. Public involvement on such a scale provides valuable real-time data, which facilitates health risk assessment and expands our understanding of the ecological dynamics of tick populations in Europe. Such initiatives have been conducted, for example, in Germany [5], the Netherlands [22], Czech Republic [10], and Hungary [13].

The main aim of our study was to decipher and map the occurrence of adult *Hyalomma* spp. ticks using the first citizen science project dedicated to monitor ticks in Poland.

## Methods

### Citizen science project

For the monitoring of the possible occurrence of adult questing *Hyalomma* spp. ticks, we implemented the dedicated citizen science project “Monster Tick—National Tick Collection.” The project started in early spring 2024. The project website (narodowekleszczobranie.pl) available in Polish and English languages, was launched in April 2024. The website provided general information on the aim of the project, the appearance and biology of *Hyalomma* spp. ticks, and two options to join the project: (1) through online reporting of the unusual tick, with photography and basic data on the site of observation and (2) directions and the postal address for sending specimens to our laboratory for the confirmation of species and further examination.

The website recorded high traffic, with several hundred thousand page views per month and tens of thousands of unique users. In addition, to increase awareness and reach a broader audience, informational flyers about the search for *Hyalomma* spp. ticks in Poland were distributed through traditional methods, including direct handing out and emailing.

The project website provided directions on proper tick photography, enabling species/sex identification, as well as instructions for safe collection and sending of ticks to the Faculty of Biology at the University of Warsaw. Participants were advised to safely remove ticks using protective measures, as outlined on the project website (https://narodowekleszczobranie.pl/pl/wyslij-kleszcza).

To attract citizens’ attention and increase awareness and engagement, the project was intensively advertised and promoted through a range of (social) media, including radio, television, and online platforms. Online submissions were reviewed, specimens identified to species/genus level and stage, and finally plotted on an interactive map by species/genus, providing real-time data on tick distribution (narodowekleszczobranie.pl). Scientists responded to all submissions, providing participants with feedback on specimens/photograph identifications and brief information about the species involved.

### Laboratory study

Tick specimens sent to the Department of Eco-Epidemiology of Parasitic Diseases were stored at the temperature of −80 °C. Specimens were photographed using a Zeiss STEMI 508 stereo microscope and identified using the key by Walker et al. [40]. Data on tick species, sex, developmental stage, as well as the collection site and host/environment of origin, were recorded in a database.

### Molecular identification of *Hyalomma* spp.

Ticks were homogenized with metal beads using a tissue homogenizer (TissueLyser II, Qiagen, Duren, Germany). Two ticks were cut in half lengthwise, and DNA was extracted from one half using the Genomic Mini AX Tissue DNA extraction kit (A&A Biotechnologies, Gdynia, Poland), following the standard protocol for animal tissues. For the other two specimens, one of the tick legs was cut off and used for DNA extraction as described above. Extracted DNA was stored at −20 °C until further analysis.

Molecular identification of *Hyalomma* species was performed by PCR amplification and Sanger sequencing of a 712 bp fragment of the mitochondrial cytochrome c oxidase subunit I (*cox1*) gene. One-step PCR was carried out using cox1F (5’-TAAACTTCAGGGTGACCAAAAAATCA-3’) and cox1R(5’-GGTCAACAAATCATAAAGATATTGG-3’) primers [41].

Reactions were performed in a volume of 20 μl, including 10 × PCR DreamTaq Green buffer (Thermo Fisher Scientific, Waltham, Massachusetts, USA), 5U DreamTaq polymerase (Thermo Fisher Scientific, Waltham, Massachusetts, USA), 0.33 mM dNTPs, 1 μM of each primer, and 2 μl of the extracted DNA. Negative controls were performed with nuclease-free distilled water, in the absence of template DNA.

PCR reactions were carried out in the following cycling conditions: primary denaturation at 95 °C for 3 min, followed by 40 cycles of the denaturation at 95 °C for 30 s, annealing at 48 °C for 30 s, and elongation at 72 °C for 1 min, followed by the final elongation step at 72 °C for 5 min and the hold step at 4 °C.

PCR products were subjected to electrophoresis on a 1.5% agarose gel and stained with Midori Green stain (Nippon Genetics, Düren, Germany). PCR products were sequenced in both directions by a private company (Genomed S.A., Warsaw, Poland) with the primers used for DNA amplification. Sequences were aligned and visually inspected using the CodonCode Aligner v. 11.0.1. software (University of Warsaw institutional license). Consensus sequences were compared with sequences deposited in the GenBank database using the BioEdit tool (http://www.ncbi.nlm.nih.gov/genbank/).

### Phylogenetic analyses

Phylogenetic analyses were performed for ~710 bp fragment of the *cox1* gene in Molecular Evolutionary Genetics Analysis version 11 (MEGA v. 11) [42]. All obtained DNA sequences were submitted to GenBank. Phylogenetic relationships were inferred using the Maximum Likelihood (ML) method on the basis of the General Time Reversible (GTR + G + I) model. The topology and branch lengths were optimized repeating the analysis 200 times with distinct randomized maximum parsimony trees. Branch support values were obtained from 1000 rapid bootstrap replicates.

### Map

The map was designed using ArcGIS (ESRI) version 10.8.2 software (institutional license purchased by the University of Warsaw, Warsaw, Poland). The map shows the distribution of *Hyalomma* spp. ticks in Poland, Germany [11], Hungary [48], and the Czech Republic [10].

## Results

Between April and November 2024, more than 500 online submissions containing tick photos were received, 11 of which were identified as adult *Hyalomma* spp. ticks (Additional file 2: Supplementary Fig. 1, Additional file 1: Supplementary Table S1). Most of the submitted images represented endemic tick species: *D. reticulatus* and *I. ricinus*. Less than 10% of online submissions featured other arthropods, such as spiders, bed bugs, or beetles. In addition, we received 65 parcels containing ticks, including four adult *Hyalomma* spp. ticks (of 11 online submissions). One of the *Hyalomma* spp. ticks was found on a horse, while the other three were not attached to a host and were noticed on the ground.

### Geographical distribution

**Fig. 1:**
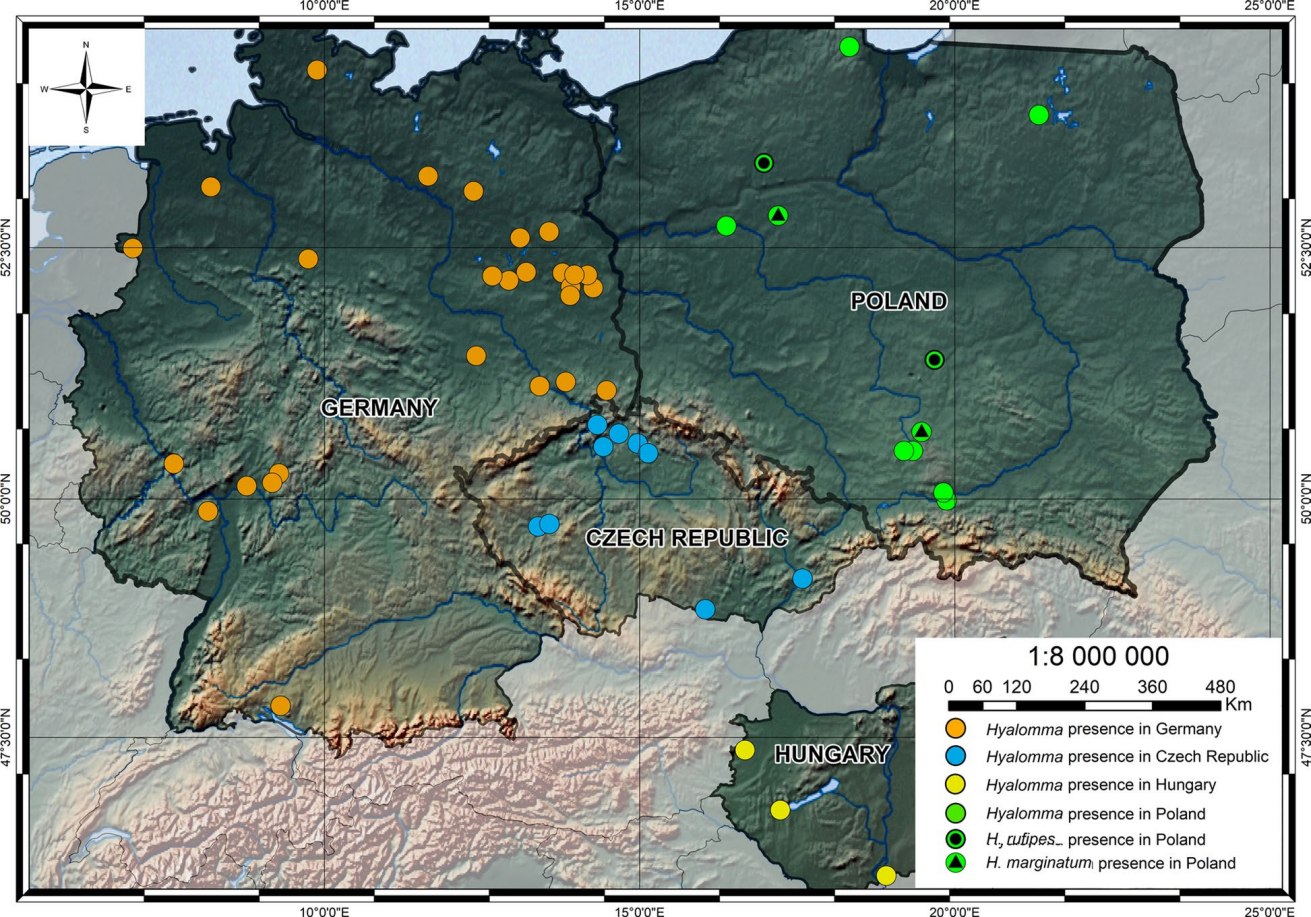
A map showing *Hyalomma* tick occurrence in Poland by reports submitted through the website narodowekleszczobranie.pl

Reports originated from six voivodeships from different regions of Poland (Additional file 1: Supplementary Table S1, Fig. 1). Most records were from Greater Poland (*n* = 3) and Silesia (*n* = 3) voivodeships, in Western and Southwestern Poland, respectively. Interestingly, two records were from one town (Poręba), along one street (Additional file 1: Supplementary Table S1).

### Morphological and molecular identification of *Hyalomma* species

Two obtained ticks were morphologically and molecularly identified as *H. rufipes*, while the other two were *H. marginatum*.

**Fig. 2:**
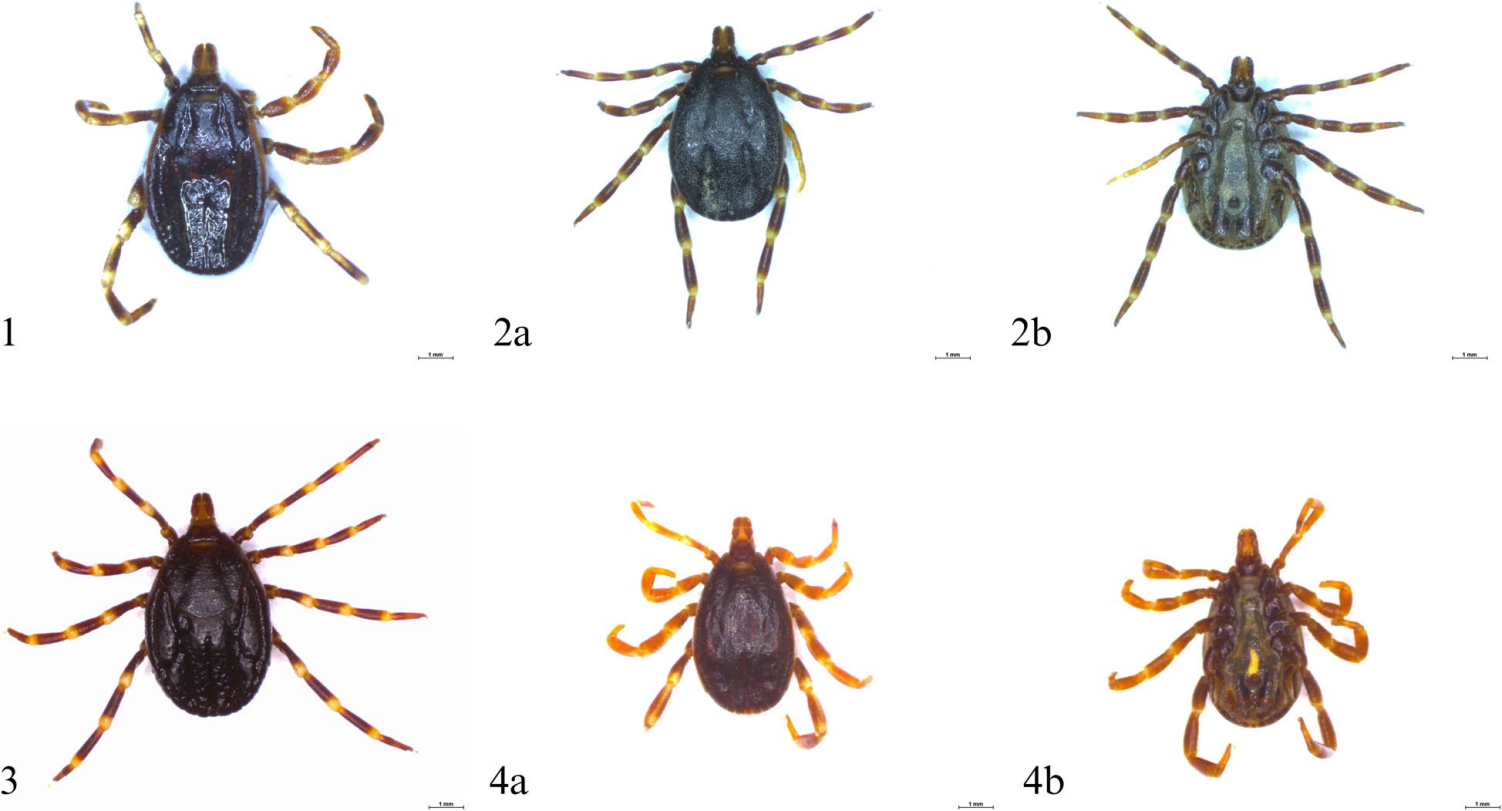
Photographs of *Hyalomma* specimens submitted through the Citizen Science project: 1. *H. marginatum*—female, 2. *H. rufipes*—female, **a** dorsal side, **b** ventral side, 3. *H. rufipe*s—female, 4. *H*. *marginatum*—male, **a** dorsal side, **b** ventral side

**Fig. 3:**
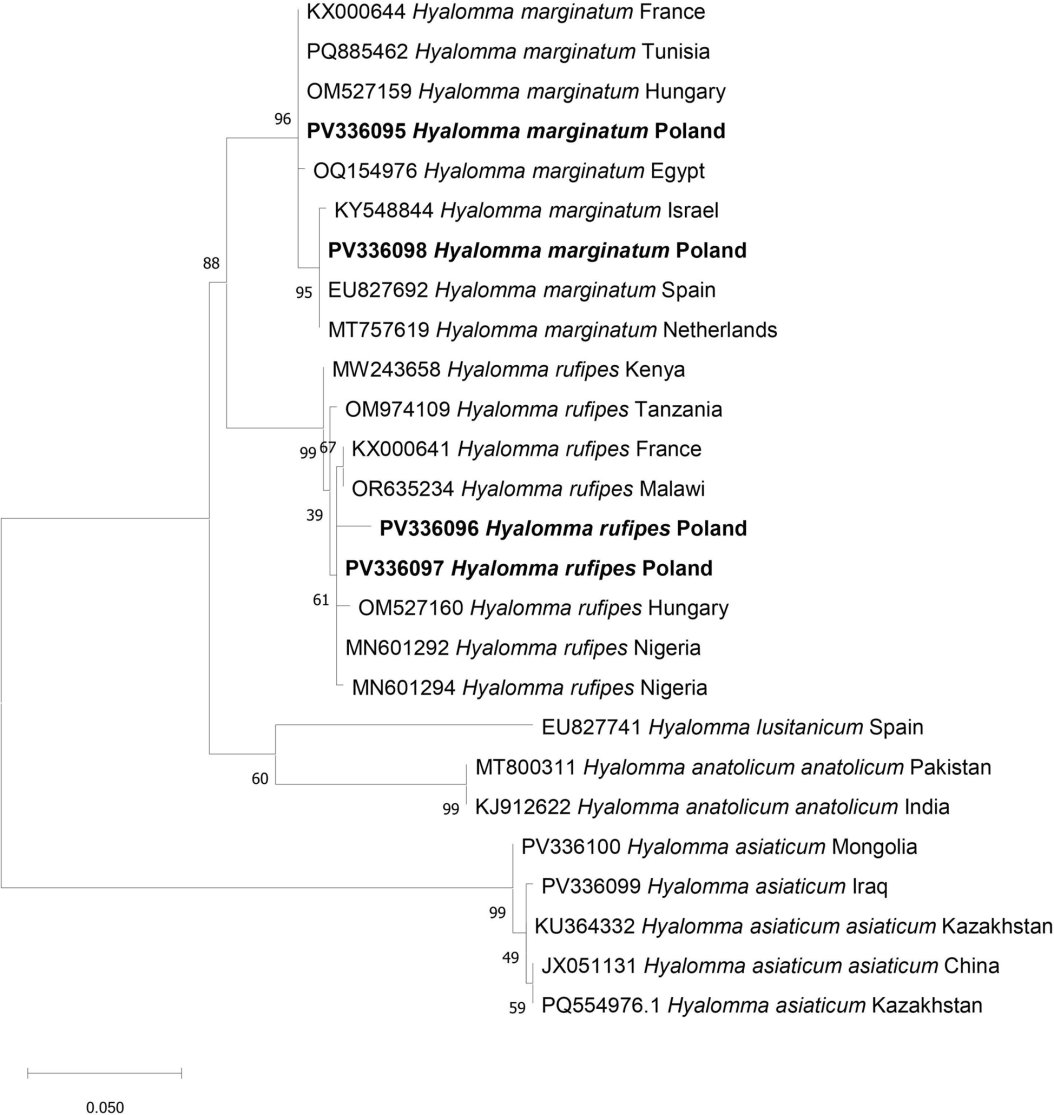
Phylogenetic analysis of *Hyalomma* spp. on the basis of *cox1* sequences (*n* = 26). The evolutionary history was inferred by using the Maximum Likelihood method on the basis of the General Time Reversible model. The percentage of trees in which the associated taxa clustered together in the bootstrap test (1000 replicates) is shown next to the branches

The first specimen (Additional file 1: Supplementary Table S1, sample no. 1), a female collected from a horse from Silesia (Śląskie voivodeship), was identified as *H. marginatum* on the basis of the morphological characteristics (Fig. 2). Identification of *H. marginatum* was confirmed by 100% *cox1* sequence identity with the sequences of *H. marginatum* from GenBank (accession number PP453758, *H. marginatum* from Tunisia). Obtained *cox1* sequence clustered on one branch with *H. marginatum* from Tunisia and other *H. marginatum* sequences (Fig. 3).

The second *H. marginatum* (sample no. 4), a male found on the ground, and also originated from Silesia (Fig. 1). The sequence of the *cox1* gene showed 100% similarity with *H. marginatum* from the Netherlands (MT757621). Furthermore, this *H. marginatum* sequence grouped with other *H. marginatum* sequences on the phylogenetic tree (Fig. 3).

The remaining two ticks, originating from Barankowo (Greater Poland = Wielkopolskie Voivodeship) and Piotrków Trybunalski (Łódzkie Voivodeship), were identified as females of *H. rufipes* on the basis of morphological features. They were characterized by a higher density of small size punctation on the scutum compared with *H. marginatum* (Fig. 2). Identification of *H. rufipes* was confirmed by 100% *cox1* sequence identity to the sequence of *H. rufipes* from Nigeria (MN601292). On the phylogenetic tree, our two *H. rufipes* sequences clustered with the *H. rufipes* sequences from Nigeria (MN601294, MN601292) and Hungary (OM527160) (Fig. 3).

## Discussion

The main achievement of our project is the first finding of adult *Hyalomma* spp. in Poland. Only in the first year of operation, our citizen science project yielded 11 reports of *Hyalomma* spp. and four specimens for molecular analysis.

To date, only two confirmed records of immature *Hyalomma* ticks have been reported in Poland. The first was described by Siuda and Dutkiewicz in 1979, and the second by Nowak and Solarz in 2010. In both cases, the specimens were collected from migratory birds—specifically, the yellow wagtail (*Motacilla flava*) and the sedge warbler (*Acrocephalus schoenobaenus*), respectively [32]. The oldest known *Hyalomma* specimens from Poland are preserved in the collections of the Upper Silesian Museum in Bytom and include one male collected in 1935 and three in 1943. These ticks were most likely collected from the environment in the Bytom area and were identified as *H. marginatum* based solely on morphological characteristics. Unfortunately, molecular confirmation was not available, limiting the certainty of their identification and their relevance for assessing the historical presence of the species in Poland [42].

Among our four specimens, two *Hyalomma* species were identified on the basis of a combination of traditional (morphology) and molecular (PCR + sequencing) methods. Both species, *H. marginatum* and *H. rufipes*, belong to the *H. marginatum* complex, which also involves *Hyalomma isaaci*, *Hyalomma turanicum*, and *Hyalomma glabrum* [42]. Taxonomic identification of ticks within this complex is challenging [43]. Both species have been reported as newly emerging in Central Europe: in Germany [11, 12], in Hungary [13], in Czech Republic [10], and the Netherlands [22]. A limitation of the current project is that identification of *Hyalomma* species on the basis of online submissions (home-made photographs of not sufficient quality and resolution) was not possible or not fully justified owing to a lack of accuracy. Thus, we plan to highlight and encourage members of the public to participate in the collection aspect of the project, sending tick specimens to our laboratory for proper identification and examination.

The majority of *Hyalomma* records were from western and south-western Poland. These records are localized in the vicinity of similar recorded clusters in neighboring countries, Germany and the Czech Republic, identified also during citizen science projects [10, 11]. Interestingly, in these countries, both *H. marginatum* and *H. rufipes* have been recorded on different occasions in the last 7 years [10, 11]. Notably, these recent reports of *Hyalomma* spp. ticks in this specific tri-border region of Poland, Germany, and the Czech Republic, may reflect the suitability of the environmental conditions for tick survival, or the occurrence of the aggregated migratory birds, or even the emergence of local tick populations, but all these possibilities require further studies. Importantly, several reports were also submitted from southern Poland, near the Polish–Slovak border; however, owing to a lack of available data on the presence of *Hyalomma* spp. ticks in Slovakia, it is not possible to conclude on the *Hyalomma* occurrence or clustering along the Polish–Slovakian border.

One of the key issues requiring further investigation is whether the observed *Hyalomma* spp. ticks indicate the presence of local populations in Poland or if they are sporadically imported from endemic regions. It seems more likely that these ticks have been imported to Poland through bird migration, given immature stages of *H. marginatum* primarily feed on passerine birds, which can transport them over long distances during seasonal migration [44–46]. In the UK, birds migrating from Africa were examined for *Hyalomma* infestations, and tick nymphs were found in 21% of those examined. A total of 68 ticks was collected from 971 birds (29 bird species) in this study, and 14 of the ticks were identified as *Hyalomma marginatum* [20]. Previous reports of *Hyalomma* spp. ticks in Poland also concerned juvenile ticks from birds [32, 33]. From an epidemiological point of view, adult questing ticks emerging from seasonally imported juvenile *Hyalomma* spp. may constitute a more serious threat than locally settled and breeding populations, because ticks arrive from countries endemic for “exotic” TBP – Tick-borne pathogens, TBD – Tick-borne diseases, CCHFV – Crimean-Congo hemorrhagic fever virus, CCHF – Crimean- Congo hemorrhagic fever, *Anaplasma marginale* and bovine anaplasmosis or exotic viruses, and due to transstadial transmission these pathogens may then be transmitted by a new generation of adults.

Delineation between settled and imported *Hyalomma* spp. ticks can be challenging. Ecologically established populations are defined as occurring and breeding in a certain place through several consecutive years and seasons. For ticks, established population are those where six or more ticks of a single life stage or more than one life stage of the tick were collected within a 12-month period (Center for Disease Control (CDC) website: Tick Surveillance Data Sets | Ticks | CDC). However, in case of regularly imported tick specimens this definition could be misleading. Regular observations of adult ticks in spring or a significant number of juvenile ticks (larvae and nymphs) on local hosts (i.e. nonmigrating birds, small mammals, livestock) could suggest the presence of a settled and breeding population. To date, one report on an unfed *H. rufipes* larva on a resident passerine bird species was described in Hungary [47]. The authors concluded that this finding, along with the presence of engorged nymphs and genetic uniformity of haplotypes, provided the first evidence of an autochthonous population of *H. rufipes* in Europe. They argued that the local development of multiple tick generations and the lack of long-distance migration during the nesting period of the host birds strongly support the establishment of this species under continental climatic conditions.

Importantly, the potential for *Hyalomma* spp. to colonize specific habitat types may be influenced by the ecological characteristics and habitat associations of the migratory bird species serving as hosts. For example, research conducted in Hungary documented the presence of *H. rufipes* on avian hosts with a strong affinity for reed-dominated habitats, such as the great reed warbler (*Acrocephalus arundinaceus*) and the sedge warbler (*Acrocephalus schoenobaenus*) [47]. These findings suggest that the occurrence of *Hyalomma* spp. in particular environments may reflect the habitat use and movement patterns of their bird hosts. Consequently, migratory birds may not only facilitate the long-distance dispersal of ticks but also play a determining role in shaping the ecological contexts in which tick populations may become established.

In our study, most records were sporadic, with only two reports from one village, Poręba, which further supports the idea that settled populations are unlikely. Although it is possible that the actual number of adult *Hyalomma* spp. ticks is higher than found in this 1-year study, these ticks are likely still too widely dispersed for effective mating and population establishment (Allee effect) [48, 49].

However, *Hyalomma* spp. were found in very different areas of Poland, including also relatively colder regions: at the sea coast or in the Warmińsko-mazurskie Voivodeship, which actually reflects their relatively good survival in current climatic conditions. Thus climate change might have already facilitated *Hyalomma* spp. spread and survival. Recent models developed by Fernández-Ruiz and Estrada-Peña [50] suggested that temperature and soil moisture are crucial in determining the conditions favorable for the development of *H. marginatum*. Their findings suggest that climate change could contribute to the emergence of new suitable habitats for this species in Europe while maintaining its endemic areas in the Mediterranean region. A spring temperature threshold of approximately + 15 °C has been identified as critical for initiating the molting process in engorged nymphs of *H. marginatum*, which is highly sensitive to ambient temperature. Laboratory studies have demonstrated that the duration of this developmental stage ranges from around 20 days at 28 °C to as long as 70 days at 18 °C [51]. Therefore, it is essential to continue research on predicting future risk areas and monitoring climate change to assess the threats associated with the spread of the *H. marginatum* complex and associated pathogens.

## Conclusions

The study successfully demonstrated that citizen science can be an effective tool for detecting and mapping the occurrence of adult *Hyalomma* ticks in Poland. The detection of both *H. marginatum* and *H. rufipes* in different parts of the country suggests that participatory surveillance may serve as a valuable addition to conventional monitoring and help in the early identification of a new tick species appearing in the region.

## Supplementary Information

Additional file 1: Supplementary Table S1 A summary of data on *Hyalomma *specimens.

Additional file 2: Fig 1 1a-c: Photographs of *Hyalomma *spp. submitted by citizens through the website narodowekleszczobanie.pl.

## Data Availability

All relevant data is presented within manuscript files (text, tables, supplementary material); sequences obtained in this study were deposited in the GenBank database.
