# Supplementary material for: The first finding of Hyalomma rufipes in Poland in 2024: the promising start of a citizen science project
Source: Parasit Vectors. 2025 Sep 24;18:383. doi: 10.1186/s13071-025-07022-4 (PMC12462190; doi:10.1186/s13071-025-07022-4)
Supplement: Supplementary file 1 — Additional file 1: Supplementary Table S1 A summary of data on Hyalomma specimens. [file 13071_2025_7022_MOESM1_ESM.docx]

| No. | Species | Male/Female | Host/Environment | Date | Location | Voivodeship | N coordinates | E coordinates | Accession No. |
| --- | --- | --- | --- | --- | --- | --- | --- | --- | --- |
| 1 | *Hyalomma* spp. | Male | Human (not attached) | May 2024 | Wronki | Wielkopolskie | 52.709798 | 16.377616 | - |
| 2 | *H. marginatum* | Female | Horse | July 2024 | Gorzków Nowy | Śląskie | 50.679558 | 19.473213 | PV336095 |
| 3 | *H. rufipes* | Female | Environment | August 2024 | Barankowo | Wielkopolskie | 53.311468 | 16.975871 | PV336096 |
| 4 | *H. rufipes* | Female | Environment (House) | August 2024 | Piotrków Trybunalski | Łódzkie | 51.401844 | 19.680897 | PV336097 |
| 5 | *Hyalomma* spp. | - | Environment | August 2024 | Kraków | Małopolskie | 49.986000 | 19.870685 | - |
| 6 | *Hyalomma* spp. | - | Horse | August 2024 | Tokary | Pomorskie | 54.400241 | 18.331384 | - |
| 7 | *Hyalomma* spp. | Male | Horse | August 2024 | Piecki | Warmińsko-mazurskie | 53.764917 | 21.335531 | - |
| 8 | *Hyalomma* spp. | - | Environment | August 2024 | Kraków | Małopolskie | 50.064991 | 19.821831 | - |
| 9 | *Hyalomma* spp. | Female | Horse | September 2024 | Poręba | Śląskie | 50.488815 | 19.335878 | - |
| 10 | *Hyalomma* spp. | - | Horse | September 2024 | Poręba | Śląskie | 50.487946 | 19.194353 | - |
| 11 | *H. marginatum* | Male | Environment | November 2024 | Wągrowiec | Wielkopolskie | 52.808511 | 17.200187 | PV336098 |
