# Supplementary figures and images for: The first finding of Hyalomma rufipes in Poland in 2024: the promising start of a citizen science project
Source: Parasit Vectors. 2025 Sep 24;18:383. doi: 10.1186/s13071-025-07022-4 (PMC12462190; doi:10.1186/s13071-025-07022-4)

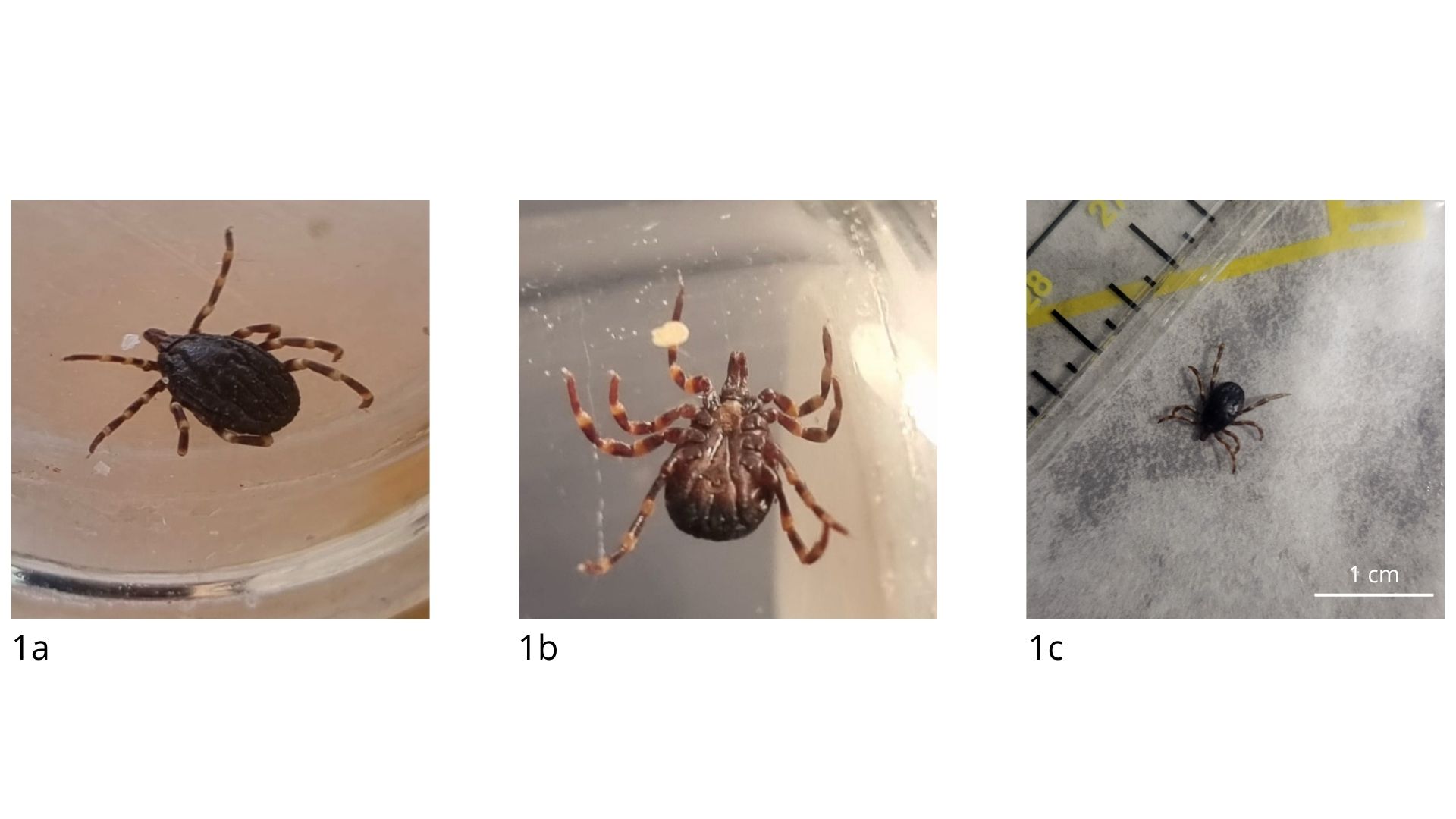

Supplement: Supplementary file 2 — Additional file 2: Fig 1 1a-c: Photographs of Hyalomma spp. submitted by citizens through the website narodowekleszczobanie.pl. [file 13071_2025_7022_MOESM2_ESM.jpg]
